# Impact of anxiety, depression and disease-related distress on long-term glycaemic variability among subjects with Type 1 diabetes mellitus

**DOI:** 10.1186/s12902-022-01013-7

**Published:** 2022-05-11

**Authors:** Alejandro Déniz-García, Alba Díaz-Artiles, Pedro Saavedra, Dácil Alvarado-Martel, Ana M. Wägner, Mauro Boronat

**Affiliations:** 1 Section of Endocrinology and Nutrition, Complejo Hospitalario Universitario Insular Materno-Infantil, Avenida Marítima del Sur, s/n. 35016, Las Palmas de Gran Canaria, Spain; 2 Institute of Biomedical and Health Research, University of Las Palmas de Gran Canaria, Las Palmas de Gran Canaria, Spain; 3 Mathematics Department, University of Las Palmas de Gran Canaria, Las Palmas de Gran Canaria, Spain

**Keywords:** Long-term glycaemic variability, Anxiety, Depression, Distress

## Abstract

**Background:**

Anxiety, depression, and disease-related distress are linked to worse overall glycaemic control, in terms of HbA1c. This study was aimed to evaluate whether traits of these emotional disorders are associated with long-term glycaemic variability in subjects with Type 1 diabetes.

**Methods:**

Longitudinal retrospective study. Six-year HbA1c data (2014–2019) from 411 subjects with Type 1 diabetes who had participated in a previous study to design a diabetes-specific quality of life questionnaire in the year 2014 were included. Scores for Spanish versions of the Hospital Anxiety and Depression Scale (HADS) and Problem Areas in Diabetes (PAID) scale were obtained at baseline, along with sociodemographic and clinical data. Long-term glycaemic variability was measured as the coefficient of variation of HbA1c (HbA1c-CV). The association between HADS and PAID scores and HbA1c-CV was analysed with Spearman correlations and multiple regression models, both linear and additive, including other covariates (age, sex, diabetes duration time, type of treatment, baseline HbA1c, use of anxiolytic or antidepressant drugs, education level and employment status).

**Results:**

Scores of depression, anxiety and distress were positively and significantly correlated to HbA1c-CV in univariate analyses. Multiple regression study demonstrated an independent association only for diabetes distress score (*p* < 0.001). Age, diabetes duration time, baseline HbA1c, education level and employment status were also significantly associated with HbA1c-CV. However, when subjects were analyzed separately in two age groups, distress scores were associated with HbA1c-CV only among those aged 25 years or older, while anxiety scores, but not distress, were associated with HbA1c-CV among those younger than 25 years.

**Conclusions:**

Psychological factors, particularly disease-related distress and anxiety, are associated with long-term glycaemic variability in subjects with Type 1 diabetes.

## Background

During recent years, a growing interest has emerged around the importance of fluctuations in blood glucose levels, collectively known as glycaemic variability, and their possible impact on diabetes clinical outcomes [1, 2]. Some studies indicate that intermittent hyperglycaemia promotes higher levels of oxidative stress and vascular and tissue damage than permanent hyperglycaemia [3] and numerous observational studies and post-hoc analyses of clinical trials have reported an association between glycaemic variability and the development of micro and macrovascular diabetes complications, severe and night-time hypoglycaemia and overall mortality [4–8]. Glycaemic variability can be assessed in short-term (within- or between-day glycaemic fluctuations) or in long-term, as variations experienced in glucose levels over weeks or months. While new continuous glucose monitoring systems have allowed the development of a great number of metrics to quantify short-term variability [1, 2], long-term glycaemic variability is usually measured as visit-to visit HbA1c oscillations [2]. On the whole, however, while much has been published about its clinical implications and the methods for its measurement, research into the potential causes of glycaemic variability and, in particular, of long-term variability, is more scarce.

Several psychological disorders, such as anxiety, depression or disease-related distress have been associated with chronic complications and worse metabolic control in subjects with both Type 1 and Type 2 diabetes [9–12]. These conditions are more frequent in subjects with diabetes than in the general population [13, 14], even more so when diagnosis is made at an early age for Type 1 diabetes [15]. The mechanisms that explain the relationship between psychological problems and diabetes complications are not fully understood, but long-term glycaemic variability could act as a putative link between both conditions, as mood and anxiety disorders can promote significant fluctuations in treatment adherence and self-care [16–18]. The aim of this study was to evaluate whether symptoms of anxiety, depression, and disease-related distress, assessed at baseline, predict long-term glycaemic variability in subjects with Type 1 diabetes over six years.

## Methods

### Participants

**Fig. 1:**
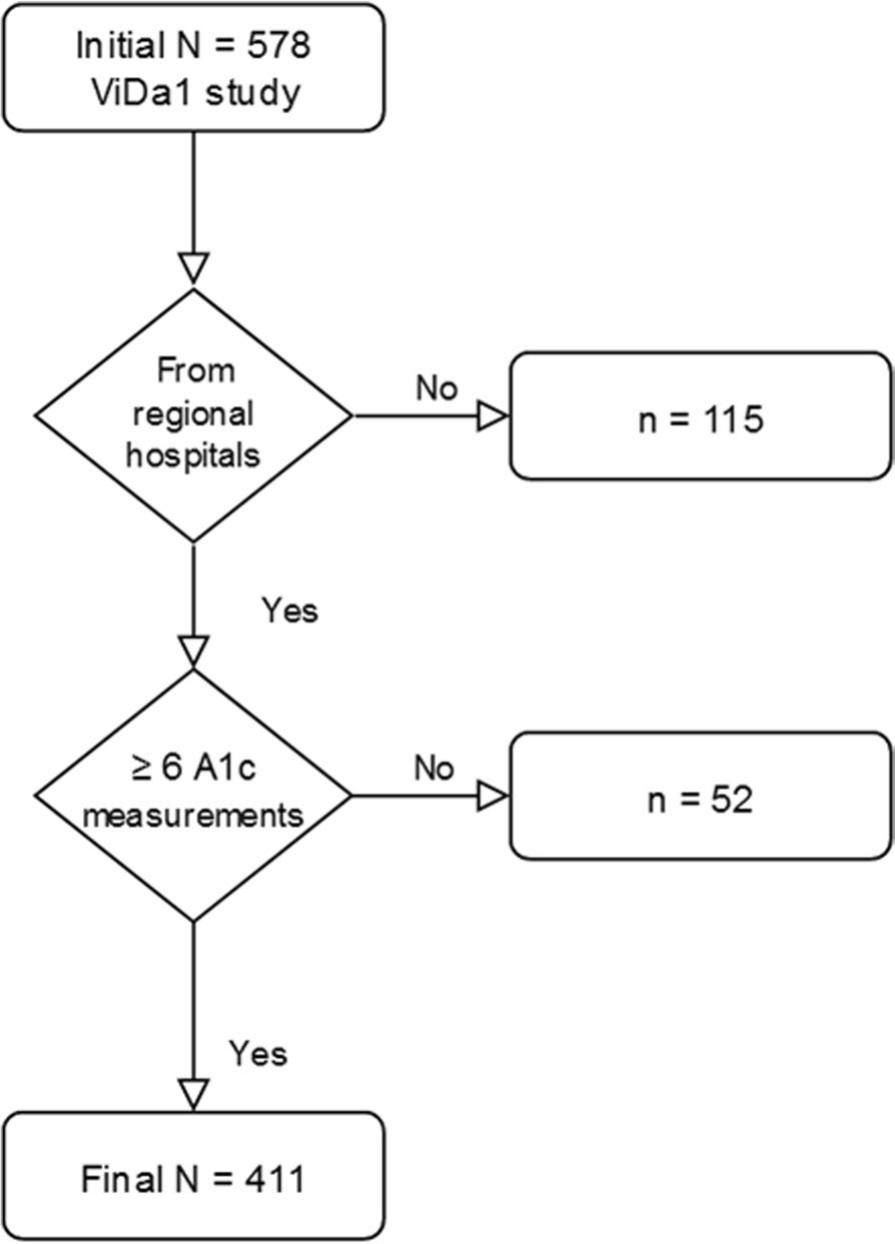
Flowchart of the study participants

Subjects were participants in a previous cross-sectional study, aimed to design and validate a questionnaire to measure quality of life in persons with Type 1 diabetes (ViDa1 questionnaire) [19]. That study was a Spanish multicentre survey conducted in 2014 in which 578 individuals over 14 years of age with Type 1 diabetes were enrolled. The only exclusion criteria for participation were pregnancy and inability to complete the study dossier because of language difficulties. Of the 578 subjects that took part in the primary study, the 463 recruited in either the Complejo Hospitalario Universitario Insular Materno-Infantil or the Hospital General Universitario Dr. Negrín, both located in Las Palmas de Gran Canaria, were included for the present investigation. Of these, in turn, 52 cases that had undergone fewer than six HbA1c determinations from their participation in the ViDa1 Study until December 2019, were excluded. Therefore, the final study population was composed of 411 subjects. Figure 1 shows the participants inclusion flow chart.

### Baseline procedures

#### Psychometric instruments

As part of their participation in the Vida1 Study, in 2014 all subjects fulfilled the Spanish versions of the Hospital Anxiety and Depression Scale (HADS) [20] and the Problems Areas in Diabetes scale (PAID) [21]. HADS is one of the most used tools to identify emotional impairment in subjects from different types of populations. It evaluates 14 items, 7 for anxiety (HADS-A) and 7 for depression (HADS-D), using a Likert scale (0–3), and the score ranges from 0 to 21 for each subscale. A cut-off point ≥ 8 on either subscale is usually considered for screening of depression or anxiety [22]. On the other hand, PAID was specifically designed to measure the emotional impact of dealing with Type 1 diabetes. It is composed by 20 items and each is answered on a Likert scale from 0 ("not a problem") to 4 ("a very serious problem"). Results of each item are added up and multiplied by 1.25, resulting in a total score from 0 to 100. Total scores of 40 or more indicate severe diabetes distress [23]. In addition, other baseline variables were recorded, including demographic data, date of diabetes onset, HbA1c level, treatment (insulin multiple daily injections or continuous subcutaneous insulin infusion), use of anxiolytic or antidepressant drugs, education level and employment situation.

### Long-term glycaemic variability

HbA1c measurements were retrieved for all subjects from the date of their participation in the Vida1 Study to December 2019. Being ${HbA1c}_{i,t}$ the HbA1c level observed in the $i$ th subject at time $t (t = 1, \dots , Ti)$ ($Ti$ varies in each subject), the variance corresponding to the underlying process is obtained by:

$${S}_{i}^{2}=\frac{1}{{T}_{i}}\sum_{t=1}^{{T}_{i}}({HbA1c}_{i,t}-{HbA1c}_{i,\circ }{)}^{2}$$

where ${HbA1c}_{i,\circ }=(1/{T}_{i}){\sum }_{t=1}^{{T}_{i}}{HbA1c}_{i,t}$. Finally, the coefficient of variation of HbA1c (HbA1c-CV) for each $i$ th subject was defined by: $HbA1c-{CV}_{i}={S}_{i}/{HbA1c}_{i,\circ }$, and was used as measure of long-term glycaemic variability. Laboratories of both hospitals used Diabetes Control and Complications Trial (DCCT)-aligned methods for HbA1c measurement throughout the whole study period.

### Statistical analysis

Categorical variables are expressed as frequencies and percentages, continuous variables as mean and standard deviation (SD) when data followed a normal distribution, or as median and interquartile range (IQR = 25th–75th percentile) when did not. The percentages were compared, as appropriate, using the Chi-square (χ^2^) test or the exact Fisher test, the means by the t-test, and the medians by the Wilcoxon test for independent data. In order to reduce skewness, HbA1c-CV data were logarithmically transformed for all statistical analyses The associations between HbA1c-CV values and the baseline psychometric measures (HADS-A, HADS-D and PAID scores) were evaluated by Spearman correlations. To assess the independent effect of psychological variables on long-term glycaemic variability, a multiple regression analysis was performed, including the results of all psychometric scales, as well as demographic and clinical variables. Selection of variables was performed using best subsets regression, a technique that consists of testing all possible combination of the predictor variables, and displays the best-fitting model. The Akaike information criterion was used to assess the goodness of fit of the models. The eventual non-linear effect of the selected numerical variables was explored using additive models. In the final model, the non-linear effects were estimated using cubic splines. The model was summarized, as appropriate, as coefficients and standard errors (SE) or cubic splines (95% confidence intervals), *P*-values and the AIC values for the model that would result when the corresponding factor was dropped. Statistical significance was set at *p* < 0.05. Data were analysed using R package, version 3.6.1.

### Ethical issues

This research project was conducted in accordance with the Helsinki Declaration, and it was approved by the Ethics Committee the Hospital General Universitario Dr. Negrín. Before entering the study, all subjects or their parents signed informed consent, with the assent of those under the age of 18 years.

## Results

### Baseline characteristics of the study subjects

Group average age was 35.5 ± 13.0 years. Out of the 411 subjects, 240 (58.4%) were women. Median diabetes duration time was 16 (10‒24) years. Women had higher scores for anxiety and depression, although statistical significance was achieved only for the anxiety score (*p* = 0.002). Also, women showed significantly higher scores in the PAID questionnaire (*p* < 0.001). Using a cut-off point ≥ 8 for the two subscales of the HADS questionnaire, 57.5% and 25.1% of participants had scores suggestive of anxiety and depression, respectively, while the PAID questionnaire revealed that up to 64.6% of them had significant diabetes-related distress. Other baseline characteristics are shown in Table 1.

**Table 1:** Baseline clinical and sociodemographic characteristics of the study participants

	Overall *N* = 411	Men *N* = 171	Women *N* = 240	P
Age, years	35.5 ± 13.0	35.1 ± 11.4	35.4 ± 12.3	0.822
Diabetes duration time, years	16 (10‒24)	14 (10‒21)	18 (11‒25)	0.057
HbA1c, %	7.8 (7.2‒8.7)	7.8 (7.2‒8.75)	7.8 (7.8‒8.7)	0.642
Use of psychotropic drugs, %	14.6	9.4	18.3	0.011
Education level, %				0.985
Primary or lower	35.0	35.1	35.0	
Secondary or tertiary	65.0	64.9	65.9	
Employment status, %				0.026
Student	18.9	15.2	20.0	
Active	49.4	57.9	43.3	
Unemployed	25.5	19.9	29.6	
Others	7.1	7.0	7.1	
Type of treatment, %				0.011
Multiple insulin injections	84.7	90.1	80.8	
Insulin pump	15.3	9.9	19.2	
HADS‒A, score	8 (6‒11)	8 (6‒10)	9 (6‒11)	0.002
HADS-A ≥ 8, %	57.5	51.5	61.8	0.029
HADS‒D, score	4 (1‒8)	4 (1‒7)	5 (1‒8)	0.304
HADS-D ≥ 8, %	25.1	21.9	27.3	0.194
PAID, score	51 (31‒68)	46 (30‒59)	55 (36‒72)	< 0.001
PAID ≥ 40, %	64.6	58.7	68.9	0.024

### HbA1c variability

**Fig. 2:**
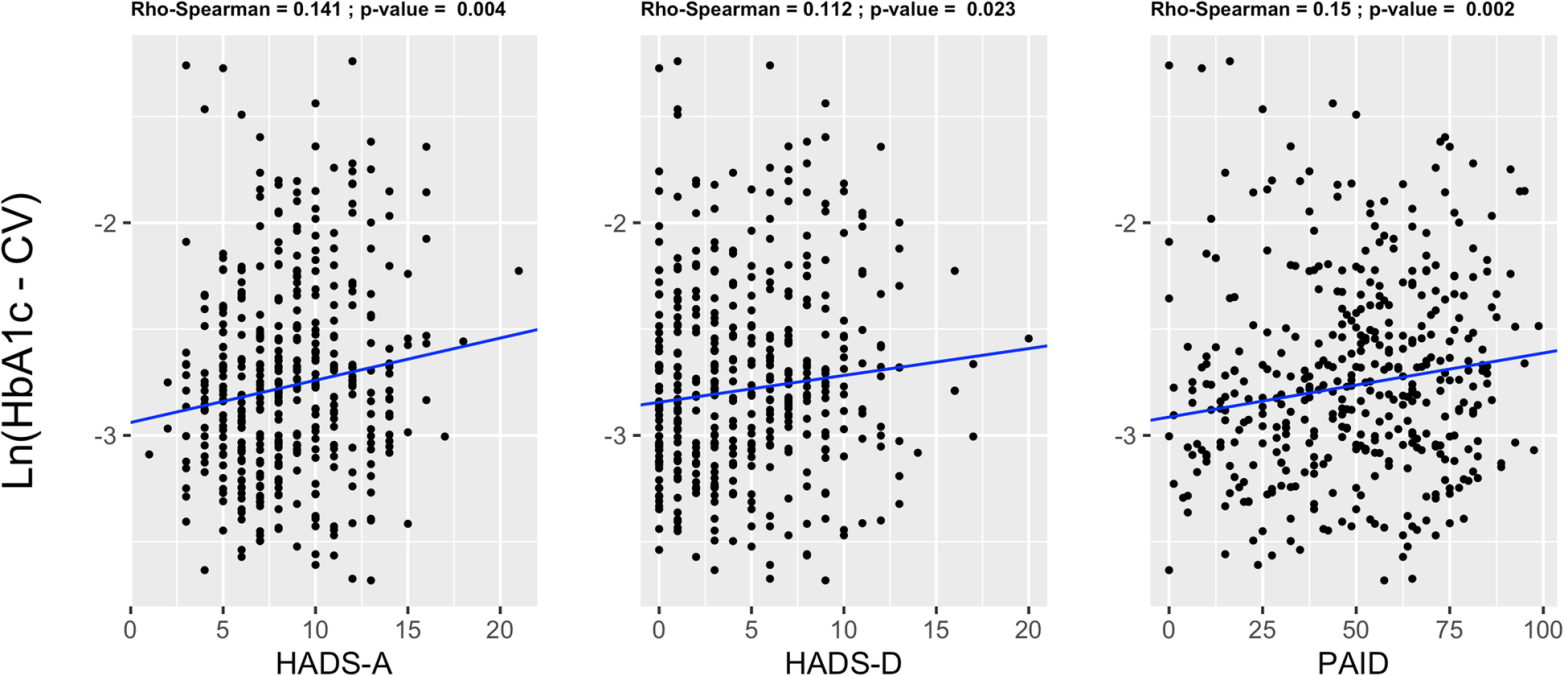
Correlations between scores of anxiety (HAD-A), depression (HAD-D), diabetes-related distress (PAID) and logarithmic HbA1c-CV

HbA1c was measured at least 6 times in a 6-year period. Spearman analyses showed significant positive correlations between HbA1c-CV and scores of HADS-A, HADS-D and PAID (*p* = 0.004, *p* = 0.023 and *p* = 0.002, respectively) (Fig. 2). In multivariate analyses, only PAID score retained an independent association with HbA1c-CV. In fact, both HADS-D and HADS-A were excluded from the model when entered simultaneously with PAID (Table 2).

**Table 2:** Multivariate regression model for logarithmic HbA1c‒CV

	Coefficient (SE)	P	AIC^a^
(Intercept)	-2.890 (0.043)	< 0.001	‒
Primary studies or lower	0.079 (0.039)	0.046	314.3
Unemployment	-0.074 (0.042)	0.077	313.7
PAID	0.003 (0.001)	< 0.001	322.4
Diabetes duration time	Cubic spline	0.004	325.3
Age	Cubic spline	< 0.001	329.7
Baseline HbA1c	Cubic spline	< 0.001	404.5

^a^*AIC* (Akaike Information Criterion) is a measure of model goodness-of-fit (the lower the AIC, the better the model fit). AIC for the full model was 312.1. Table shows AIC values after removing any of the selected variables

**Fig. 3:**
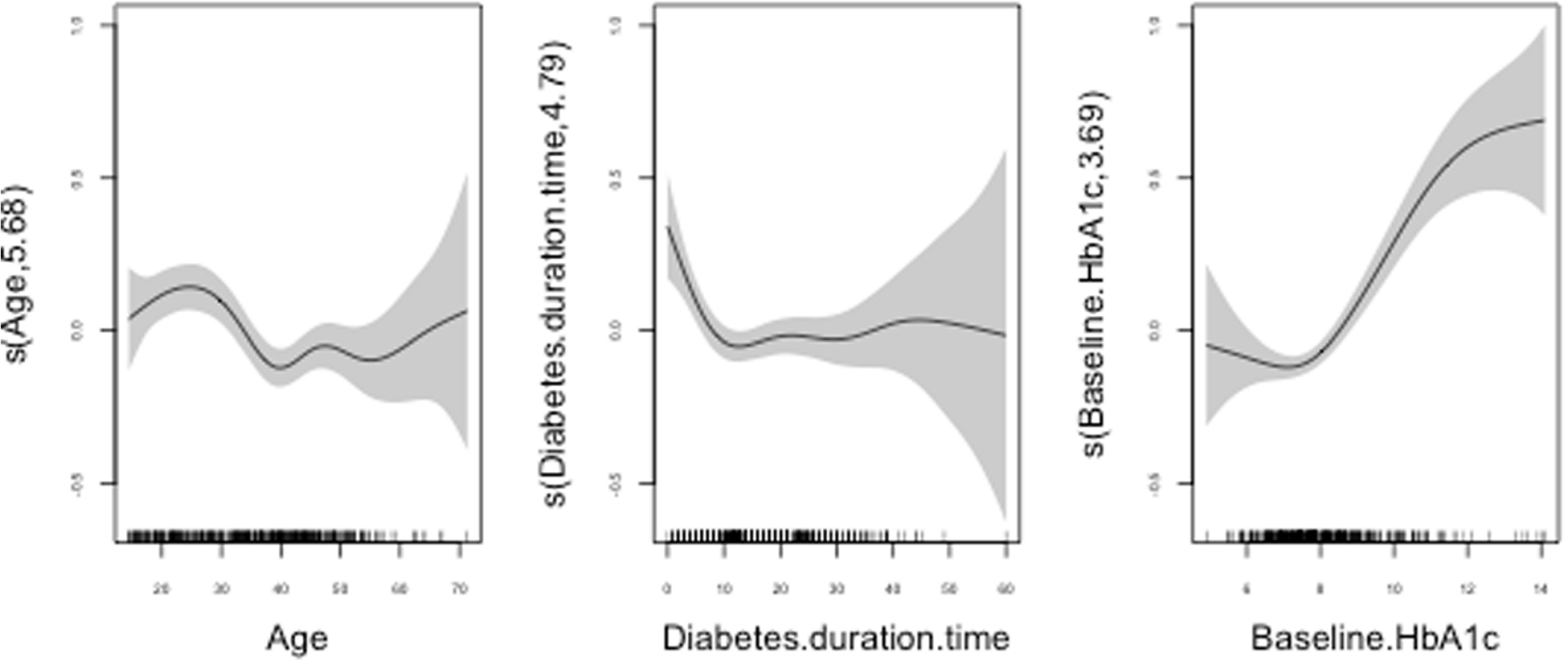
Non-linear effects of age, diabetes duration time and baseline HbA1c on logarithmic HbA1c-CV (95% CI)

Regarding to the rest of assessed covariates, lower education level (positively) and unemployment (negatively) were also linearly correlated to long-term glycaemic variability, while the additive regression models showed non-linear effects of age, diabetes duration time and baseline HbA1c on HbA1c-CV. In relation to the latter, as shown in Fig. 3, HbA1c-CV increased with age up to 20 years and decreased thereafter, stabilising at around 40 years. Similarly, long-term glycaemic variability was also highest in the early stages of the disease, falling and stabilising after 10 years of diabetes duration. Finally, HbA1c-CV was observed to rise abruptly when participants' baseline HbA1c levels increased above 8%.

**Fig. 4:**
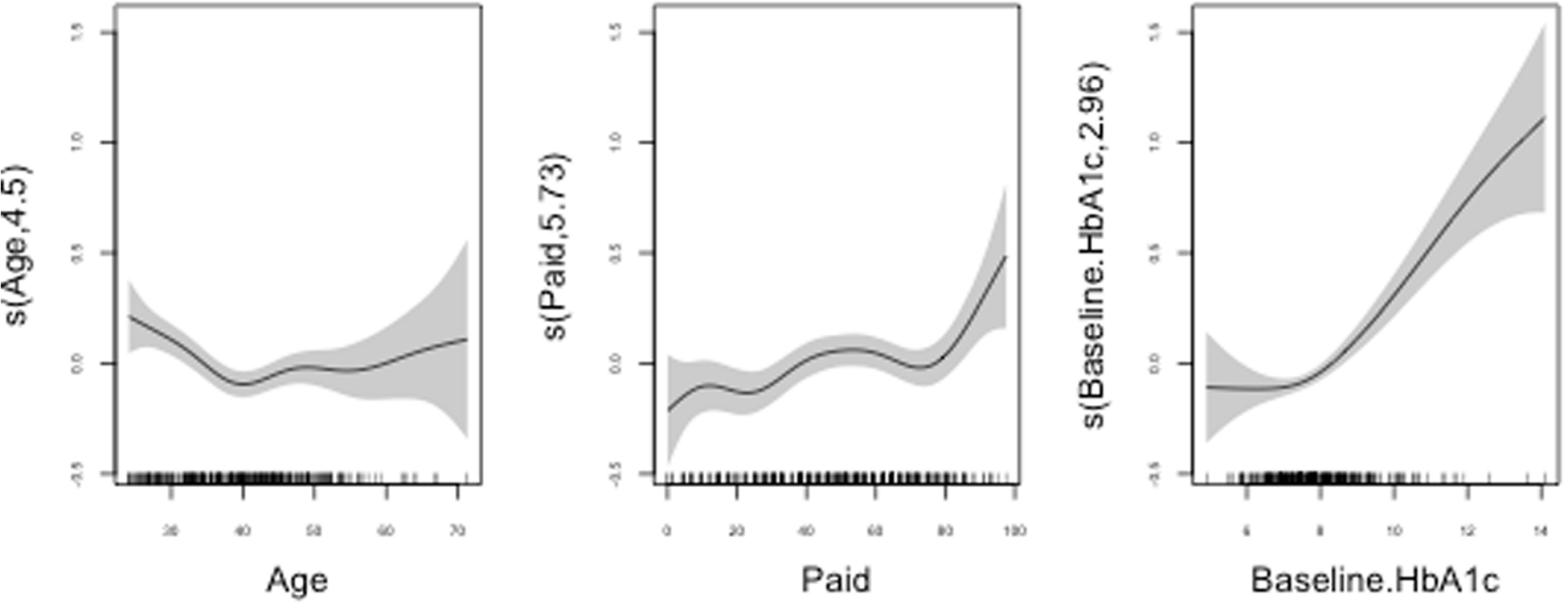
Non-linear effects of age, diabetes-related distress (PAID) and baseline HbA1c on logarithmic HbA1c-CV (95% CI) among subjects 25 years of age or older

**Fig. 5:**
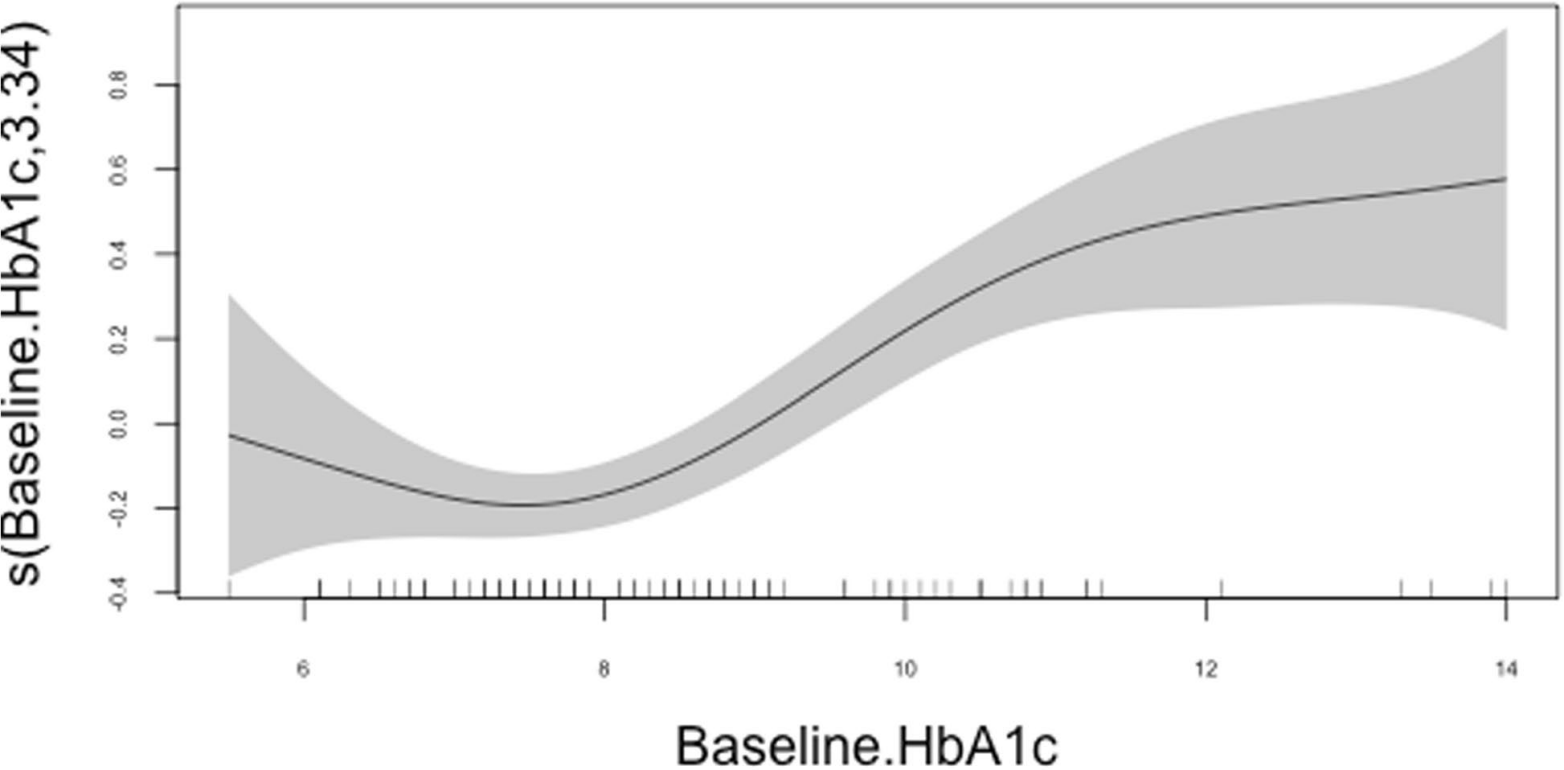
Non-linear effect of baseline HbA1c on logarithmic HbA1c-CV (95% CI) among subjects younger than 25 years

Given the effect of age on HbA1c-CV, two separate regression models were further constructed for the 94 participants up to 24 years of age (a limit recently proposed to define the life span comprising adolescence) [24] and for those aged 25 years or older (Table 3). Among the latter, an independent association remained between HbA1c-CV and baseline HbA1c, age, and PAID score, although in this case the correlation with HbA1c-CV was nonlinear in all cases, including PAID (Fig. 4). In contrast, among adolescents, the best model was composed of baseline HbA1c (non-linearly, represented in Fig. 5), duration of diabetes and HADS-A score, but not PAID.

**Table 3:** Multivariate regression models for logarithmic HbA1c‒CV, according to age groups

	Age > 24 years (*N* = 317)			Age ≤ 24 years (*N* = 94)		
	Coefficient (SE)	P	AIC^a^	Coefficient (SE)	P	AIC^†^
(Intercept)	-2.810 (0.020)	< 0.001	‒	-2.473 (0.131)	< 0.001	‒
Age	Cubic spline	0.004	268.9			
PAID	Cubic spline	0.001	273.2			
Baseline HbA1c	Cubic spline	< 0.001	327.9	Cubic spline	< 0.001	94.8
Diabetes duration time				-0.019 (0.008)	0.013	66.9
HADS-A				0.017 (0.010)	0.099	62.8

^a^*AIC* (Akaike Information Criterion) for the full model was 257.8. ^†^AIC for the full model was 62.3. Table shows AIC values after removing any of the selected variables

## Discussion

The main finding of this study is that, in subjects with Type 1 diabetes, higher levels of emotional disturbances, mainly disease-related distress and anxiety, are related to greater long-term HbA1c variability. Separate analysis of individuals by age group showed that anxiety scores had a greater impact on visit-to visit HbA1c variability in adolescents and young adults, whereas in subjects 25 years or older the main psychological determinant of glycaemic variability was disease-specific distress. Depression traits, although strongly correlated to HbA1c-CV in univariate analysis, were no longer associated with the outcome in the multivariate models. Age, duration of diabetes, employment status and educational attainment were also associated with glycaemic variability in the whole sample. The effects of age and duration of diabetes were non-linear, and could be expected. So, long-term glycaemic variability was greater in younger patients, in whom it is well-known that diabetes tends to be more unstable [25], and in those with a shorter duration of the disease, probably reflecting the loss of beta cell function and the progressive deterioration of glycaemic control during the first years of diabetes course [26]. In fact, in separate analyses by age group, duration of diabetes was only significantly associated with HbA1c-CV among those under 25 years of age. The negative correlation between HbA1c-CV and unemployment status was more intriguing, but could be related to previous observations showing that work-related stress is common among workers with Type 1 diabetes, and it may negatively influence metabolic control [27].

Because of its potential role as a causal agent of diabetes complications, during the last years there has been an increasing interest in the quantification and the search for strategies to reduce glycaemic variability. Some pharmacological and non-pharmacological approaches aimed at minimizing glycaemic variability have been proposed [2]. In persons with Type 1 diabetes, such interventions have been mainly based on providing more stable insulin regimens, in order to reduce fluctuations in blood glucose levels. Continuous glucose monitoring and continuous subcutaneous insulin infusion have already shown benefits in reducing short- and long-term variability [28–30]. However, although focus has been mainly placed on treatment-derived variables, it seems plausible that emotional factors could be implicated on glycaemic variability. Psychological stress promotes activation of the hypothalamic–pituitary–adrenal axis and the autonomic nervous system, eliciting the release of counter-regulatory hormones [31]. In addition, it can lead to mood and behaviour fluctuations that negatively affect self-management and treatment adherence [32]. A large number of studies indicate that affective and anxiety disorders and distress negatively influence the metabolic control of subjects with Type 1 diabetes, as globally assessed by HbA1c levels [10, 11, 15]. Nonetheless, the specific association between psychological disturbances and glycaemic variability has not received much attention. Some studies have examined the correlation between mood and short-term glycaemic variability in Type 1 diabetes, some of them evaluating different continuous glucose monitoring-derived metrics, but study samples have been small and results were inconsistent [33]. To our knowledge, the present report represents the first approach to specifically investigate on the relationship between psychological factors and long-term glycaemic variability.

The study has some significant limitations. Only an initial evaluation of the anxiety, depression, and distress levels was conducted. Their scores could have changed during the subsequent 6-year period in which HbA1c was analysed, thus undermining our ability to assess its relationship with long-term glycaemic variability. However, although not much information is available, some data suggest that affective disorders and distress persist over time in subjects with diabetes and, in addition, diabetes-related distress and depressive symptoms are independent risk factors for each other over time [34]. Secondly, given the retrospective nature of the study, although only individuals in whom at least 6 HbA1c measurements were included, the number and frequency of measurements over the 6 years of the study was not uniform and this could also affect the results. Among the study strengths, the sample size was reasonably large. Secondly, it is also noteworthy that anxiety and depression were studied together with disease-related distress. Multivariate analysis, which also adjusted for the effect of other covariates potentially linked to long-term glycaemic variability, allowed the impact of each of these variables to be ascertained separately. This may be important, because anxiety and depression are highly interrelated with the distress generated by the disease itself [35–37], although their specific influence on glycaemic control may be different. In fact, consistent with our results, other studies using multiple regression analyses in Type 1 diabetes have found that the impact of depression and anxiety symptoms on HbA1c is no significant when analysed together with disease-related distress [36–38].

## Conclusions

Psychological factors, mainly disease-related distress and anxiety, are associated with higher long-term HbA1c oscillations in subjects with Type 1 diabetes. Further studies are needed to assess whether the control of emotional disturbances can help to reduce long-term glycaemic variability and to improve diabetes related outcomes.

## Data Availability

The datasets generated during the current study data contain sensitive information and are not publicly available to avoid potential identification of participants, but are available from the corresponding author on reasonable request.

## Abbreviations

HADS: Hospital Anxiety and Depression Scale
HADS-A: Hospital Anxiety and Depression Scale-Anxiety
HADS-D: Hospital Anxiety and Depression Scale-Depression
HbA1c-CV: Coefficient of variation of HbA1c
PAID: Problems Areas in Diabetes scale
